# Transcutaneous electrical nerve stimulation over acupoint for chronic obstructive pulmonary disease: A systematic review and meta-analysis

**DOI:** 10.3389/fpubh.2022.937835

**Published:** 2022-10-06

**Authors:** Ying Wei, Nairong Yuan, Yan Dong, Lixia Wang, Jiru Ding

**Affiliations:** ^1^Experimental Management Center, Shanxi University of Chinese Medicine, Jinzhong, China; ^2^Respiration Department, Hospital of Chengdu University of Traditional Chinese Medicine, Chengdu, China; ^3^Department of Chinese Medicine, Shanxi Provincial People's Hospital, Taiyuan, China

**Keywords:** transcutaneous electrical nerve stimulation (TENS), acupoint, COPD, efficacy, meta-analysis

## Abstract

**Background:**

Transcutaneous electrical nerve stimulation over an acupoint (acu-TENS), a new technique applied in pulmonary rehabilitation programs, has been gradually used in the management of chronic obstructive pulmonary disease (COPD). However, the effects of acu-TENS have not been fully evaluated. Therefore, this review was conducted to assess the effects of acu-TENS on COPD.

**Methods:**

A total of seven electronic databases were searched from their inception to September 2021 for randomized controlled trials of acu-TENS for COPD. Two investigators independently performed data extraction and methodological quality assessment. Heterogeneity was examined by Cochrane χ^2^ and *I*^2^ tests. The source of heterogeneity was investigated by subgroup analysis or sensitivity analysis.

**Results:**

In our review, ten studies between 2008 and 2021 were included. The aggregated results indicated that acu-TENS showed positive effects in forced expiratory volume in 1 s (FEV1) [MD = 0.13 L, 95% CI (0.11–0.16), *P* < 0.00001], FEV1% predicted [MD = 5.92%, 95% CI (3.43–8.41), *P* < 0.00001], 6-min walk distance (6MWD) [MD = 14.68m, 95% CI (6.92–22.44), *P* = 0.0002], dyspnea visual analog scale (DVAS) [MD = −7.58, 95%CI (−14.33 to −0.84), *P* = 0.03], modified Borg scale (MBS) [MD = −0.46, 95% CI (−0.86 to −0.06), *P* = 0.03], and COPD assessment test (CAT) [MD = −4.25, 95% CI (−5.24 to −3.27), *P* < 0.00001]. Although six studies reported adverse effects, only one patient had shoulder pain after acu-TENS.

**Conclusion:**

Acu-TENS seems to be effective in improving pulmonary function and health status in patients with COPD, with little effect on exercise capacity and dyspnea. However, this result should be interpreted with caution, and high-quality RCTs were needed for further verification.

## Introduction

Chronic obstructive pulmonary disease (COPD) is a debilitating chronic illness characterized by sustained airflow obstruction and recurrent respiratory symptoms. Statistically, 3.2 million people died of COPD in 2015, making it the third leading cause of death worldwide (1). Therefore, effective prevention, management, and treatment of COPD remain one of the major tasks for public health worldwide. In the therapy of COPD, a pulmonary rehabilitation program is regarded as a hallmark of treatment for all patients (2). Evidence suggests that it can improve exercise capacity and reduce both fatigue and dyspnea in patients with COPD (3, 4).

Transcutaneous electrical nerve stimulation over acupoints (acu-TENS) is a new technique used in pulmonary rehabilitation programs (5). It combines acupoint stimulation therapy with transcutaneous electrical nerve stimulation (TENS), which has similar analgesic effects and peripheral and central effects as acupuncture (6). Unlike traditional acupuncture, acu-TENS is a noninvasive, easy-to-apply intervention that induces therapeutic effects through the application of surface electrodes over the specific acupoints instead of needles (5).

In recent years, acu-TENS has been gradually applied to the treatment of patients with COPD. Limited evidence suggests that acu-TENS can improve the dyspnea of patients with COPD during walking, especially those with more severe dyspnea during peak exercise (7). In addition, Shirley et al. (8) found that acu-TENS can improve the expiratory flow rates after exercise in healthy subjects. However, most of the reports on the intervention effect of acu-TENS on COPD are clinical trials with small sample sizes, which make it difficult to draw reliable conclusions. Considering that the evidence for acu-TENS in patients with COPD has not been systematically reviewed, this meta-analysis aims to analyze the clinical efficacy of acu-TENS and provide certain guidance for clinical application and further research.

## Methods

This systematic review and meta-analysis were performed following the guidelines of PRISMA (9).

### Search strategy

Literature retrieval was performed by two researchers independently. To capture as many studies as possible, the following electronic databases were searched: PubMed, Cochrane Library, Embase, Web of Science, China Network Knowledge Infrastructure (CNKI), Wanfang Database, and VIP Database. All databases were searched from inception to September 2021. Literature references were also checked for the existence of eligible studies. In terms of retrieval strategy, we adopted a combination of medical subject heading (MeSH) terms and free words. There were no restrictions on language or publication status.

### Study selection

The selection of studies was performed independently by two researchers, and any disagreements arising were resolved through discussion. In this review, the included studies met the following criteria: (a) randomized controlled trials (RCTs). (b) Participants were diagnosed with COPD, and there were no restrictions on COPD type, GOLD grade, age, gender, or nationality. (c) Acu-TENS was used as an intervention. Thus, the control interventions included conventional treatment for COPD, placebo TENS (i.e., transcutaneous electrical nerve stimulation over an acupoint without current output), or sham TENS (i.e., transcutaneous electrical nerve stimulation over a non-acupoint). In the treatment group, a combination of acu-TENS and control interventions, or acu-TENS alone, were allowed. (d) The outcome indicators included pulmonary function index assessed by the forced expiratory volume in 1 s (FEV1) and FEV1% predicted; exercise capacity assessed by 6MWD; health status assessed by CAT; and dyspnea assessed by MBS and DVAS. Literature retrieved from databases was imported into Endnote X7. By assessing the titles, abstracts, and full texts, eligible studies were included.

### Data extraction

Data extraction was performed independently by two researchers based on a pre-formulated data extraction form, and any discrepancies were resolved by discussion. The following information was extracted: first author, publication date, and country; the number of participants; duration of disease; COPD type; GOLD grade; interventions of the treatment group; interventions of the control group; acupoint; parameters of TENS; course of treatment; outcome indicators; safety; and funding. If necessary, the authors were contacted by email for relevant information.

### Quality assessment

The methodological quality of the included studies was assessed by two researchers independently using the revised Cochrane risk of bias tool (RoB 2) (10). Any objections raised by the two reviewers were solved through discussion. The risk of bias was evaluated *via* process randomization, deviations from intended interventions, missing outcome data, measurement of outcome, and selection of reported results. The study was considered “low risk of bias” only if all five aspects were assessed as low risk. However, the study was considered to have “some concerns of bias” if at least one item was judged to have some concerns. The study was considered to be a “high risk of bias” if at least one item was judged as high risk.

### Data synthesis and analysis

Data were analyzed using Review Manager 5.3. There are two methods for meta-analysis of continuous variables: inverse-variance fixed effect method and inverse-variance random-effect method. The effect estimation of continuous variables was represented by mean difference (MD) and 95% confidence interval (*CI*). Heterogeneity was examined by Cochrane *X*^2^ and *I*^2^ tests. A fixed effect model was adopted if the heterogeneity was not significant; otherwise, a random-effect model was adopted (11). The source of heterogeneity was investigated by subgroup analysis or sensitivity analysis. In addition, if there were 10 or more RCTs, publication bias was tested by funnel plots and Egger's tests.

## Results

### Search results and study characteristics

**Figure 1:**
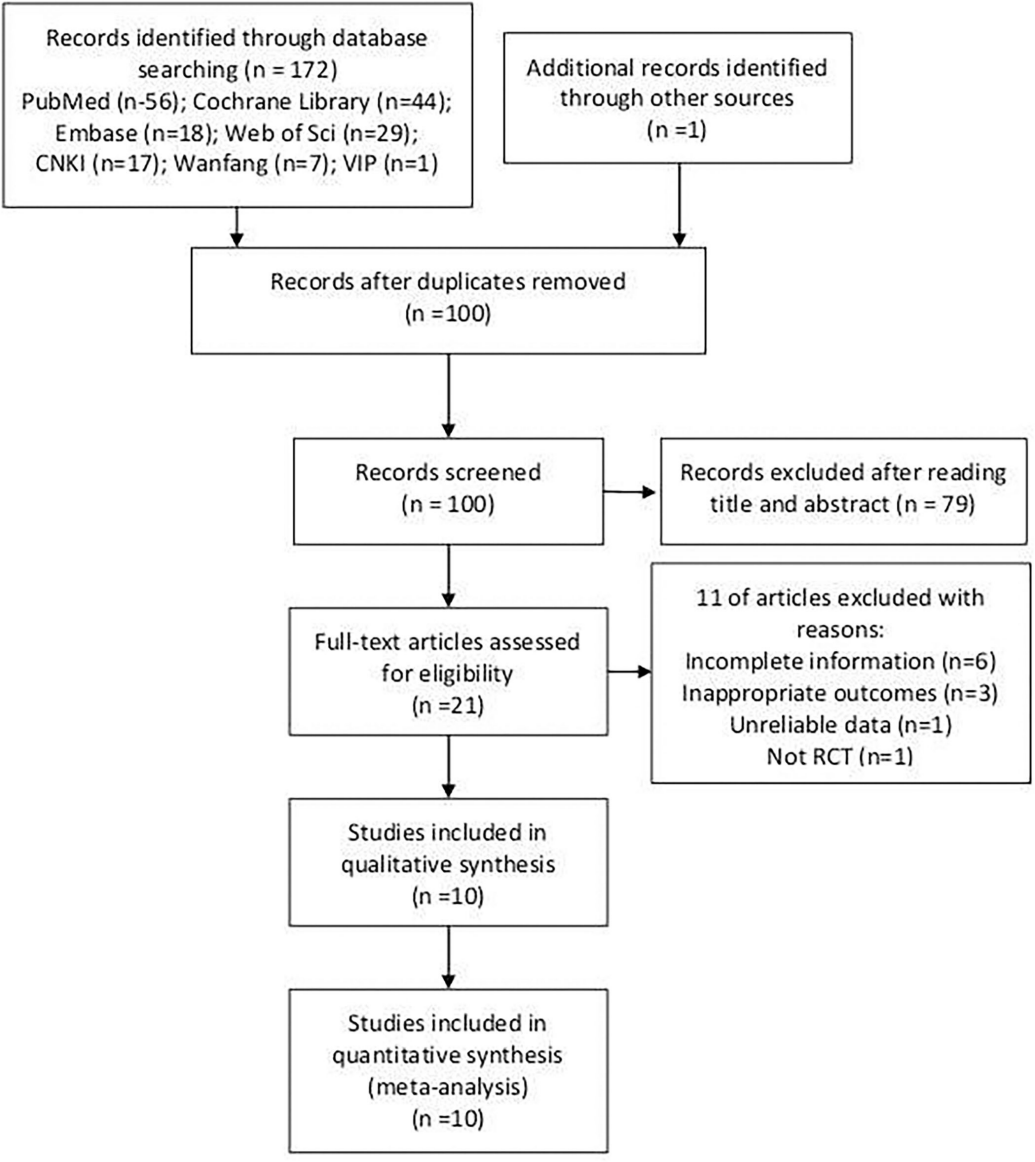
Flow chart of literature screening.

A total of 172 studies have been searched from seven databases, and 1 eligible study was found in the literature references. After removing replicates, 100 potentially relevant studies were identified. A total of 79 studies were deleted after reading the titles and abstracts. The full text of the remaining 21 studies was assessed, and 11 studies were excluded according to the inclusion criteria or the exclusion criteria. Finally, 10 studies were included in this meta-analysis (12–21). The detailed process is depicted in Figure 1.

### Characteristics of the included studies

A total of 472 patients were included, including 233 in the treatment group and 239 in the control group. The studies, published between 2008 and 2021, were conducted mainly in China, India, and Turkey. The age of patients with COPD ranged from 42 to 85 years, with an average course of 5–42 years. Among the studies, eight included patients with stable COPD, one included patients with acute exacerbation of COPD, and one did not mention the stage of COPD. In terms of acupoints, five studies applied TENS over EX-B1, one applied over BL-13, and four applied over multiple acupoints. In terms of the course of treatment, three studies received a single session of acu-TENS, and the remaining studies received multiple sessions of acu-TENS. The specific characteristics of included studies are presented in Table 1.

**Table 1:** Characteristics of included studies.

**Author, year**	**No. of patients (T/C)**	**COPD Type**	**GOLD grade**	**Intervention of treatment**	**Intervention of control**	**Acupoint**	**Course of treatment**	**TENS parameter**	**Outcome**	**Safety**
Vyas et al. (14)	28/27	Stable COPD	I or II	Basic treatment + Acu-TENS	Basic treatment + Placebo TENS (A plastic film with no central pore was placed on the acupoints so that no current was transmitted)	EX-B1	Single session for 45 min	Frequency−4 Hz; Pulse Width−200 μs	②③④	No adverse effects
Jones et al.(21)	22/22	NR	III or IV	Basic treatment + Acu-TENS	Basic treatment + Placebo TENS (The electrical output was disconnected inside the device)	EX-B1	Single session for 45 min	Frequency−2 Hz; Pulse Width−200 μs	①④	No adverse effects
Lau and Jones (20)	23/23	Stable COPD	I or II	Basic treatment + Acu-TENS	Basic treatment + Placebo TENS (A plastic film with no central pore was placed on the acupoints so that no current was transmitted)	EX-B1	Single session for 45 min	Frequency−4 Hz; Pulse Width−200 μs	①④	No adverse effects
Liu et al. (19)	25/25	Stable COPD	II, III or IV	Basic treatment + Acu-TENS	Basic treatment + Placebo TENS (Identical to Acu-TENS but with no electrical output from the machine)	EX-B1, BL-13, BL-23, ST-36	40 min every 2 days for 4 weeks	Frequency−2 Hz; Pulse Width—NR	②③④⑥	No adverse effects
Ngai et al. (18)	10/8/10	Stable COPD	III or IV	Basic treatment + Acu-TENS	Basic treatment + Placebo TENS (Identical to Acu-TENS but with no electrical output from the machine)	EX-B1	45 min per session, 5 sessions per week for 4 weeks	Frequency−2 Hz; Pulse Width−200 μs	①③	No adverse effects
Öncü and Zincir (17)	35/35	AECOPD	NR	Basic treatment + Acu-TENS	Basic treatment + Placebo TENS (The electrical output was disconnected inside the device)	Ex-B1 and Lu 7	45 min per session, total of 20 sessions	Frequency−4 Hz; Pulse Width−200 μs	①③	One patient shoulder pain
Vinod et al. (15)	15/15	Stable COPD	II	Acu-TENS	Placebo TENS (A plastic film with no central pore was placed on the acupoints so that no current was transmitted)	EX-B1	45 min per session for 1 week, total of 5 sessions	Frequency−4 Hz; Pulse Width−200 μs	①	NR
Shou et al. (16)	15/15	Stable COPD	I or II	Acu-TENS	Placebo TENS (Identical to Acu-TENS but with no electrical output from the machine)	BL-13	40 min per session, 5 days a week for 4 weeks	Frequency−4 Hz; Pulse Width−200 ms	①	NR
Xie and Li (12)	35/35	Stable COPD	II or III	Basic treatment + Acu-TENS	Basic treatment + sham TENS (TENS was performed at non-acupoints)	BL-13, LU-1	Once a day for 20 min for 2 months	Frequency−100Hz; Pulse Width−200 ms	①③	NR
Xiao et al. (13)	25/24	Stable COPD	I, II, III, VI	Basic treatment + Acu-TENS	Basic treatment + Placebo TENS (Identical to Acu-TENS but with no electrical output from the machine)	BL-13, BL-23, EX-B1, ST-36	Once every other day for 40 min for 4 weeks	Frequency−2 Hz; Pulse Width—NR	②③⑥	NR

NR, not reported; ①, FEV1; ②, FEV1% predicted; ③, 6MWD; ④, DVAS; ⑤, MBS; ⑥, CAT.

### Methodological quality

**Figure 2:**
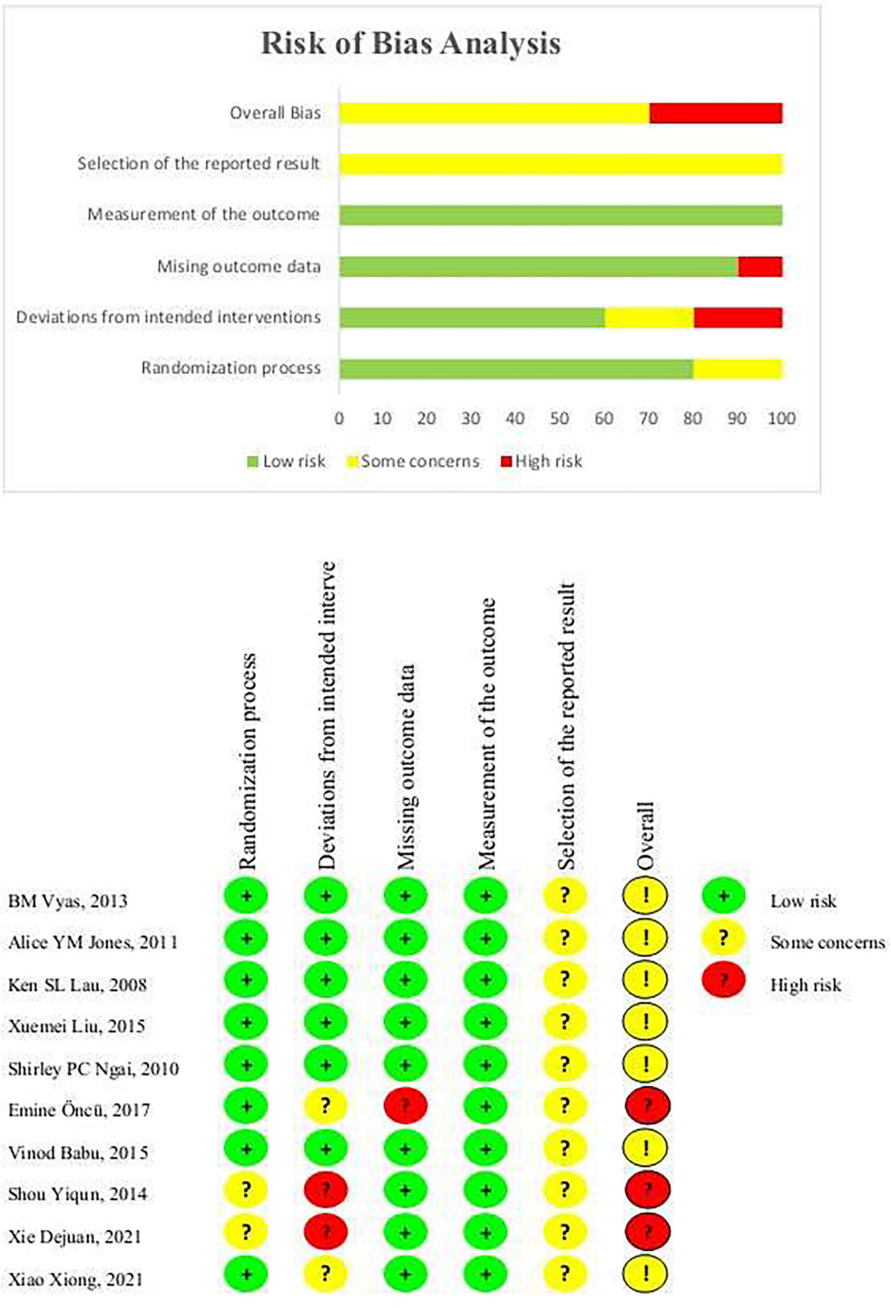
Risk of bias of included studies.

As shown in Figure 2, three studies had a high risk of bias (12, 16, 17), and seven showed some concern about bias (13–15, 18–21). Except for two studies (12, 16), the remaining studies reported the method of random sequence generation and the process of assigning concealment in detail. Since the vast majority of subjects completed the intervention, two studies (13, 17) showed deviations from the intended interventions and were judged to be of “some concerns”. In terms of missing outcome data, only one study (17) had < 90% of available outcome data. For the selective report, the judgment could not be made since none of the included trials were publicly registered or published their protocols. In summary, the missing outcome data, deviations from the intended intervention, and the selection of reported results were the main factors that limited the quality of studies.

### Synthesis of the results

#### FEV1

**Figure 3:**
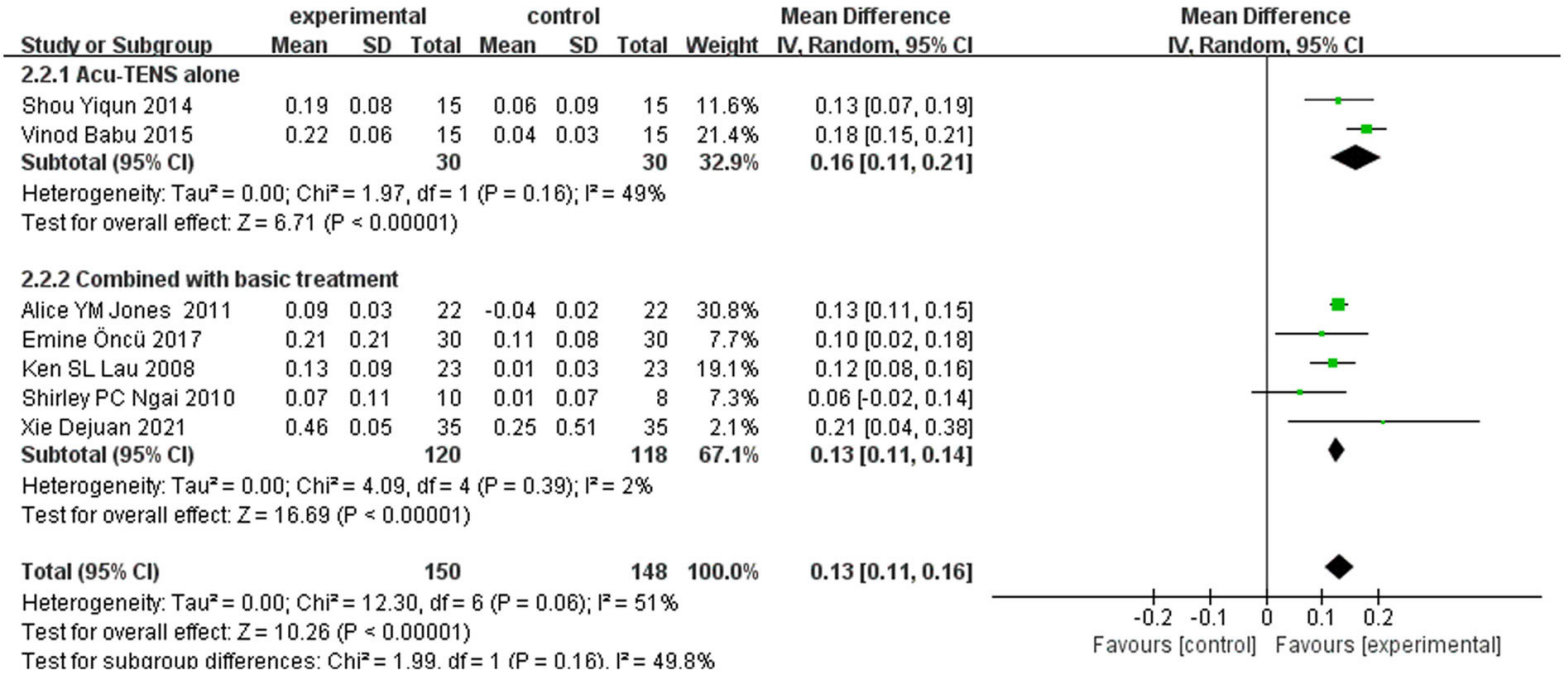
Subgroup analysis of FEV1 (intervention).

**Figure 4:**
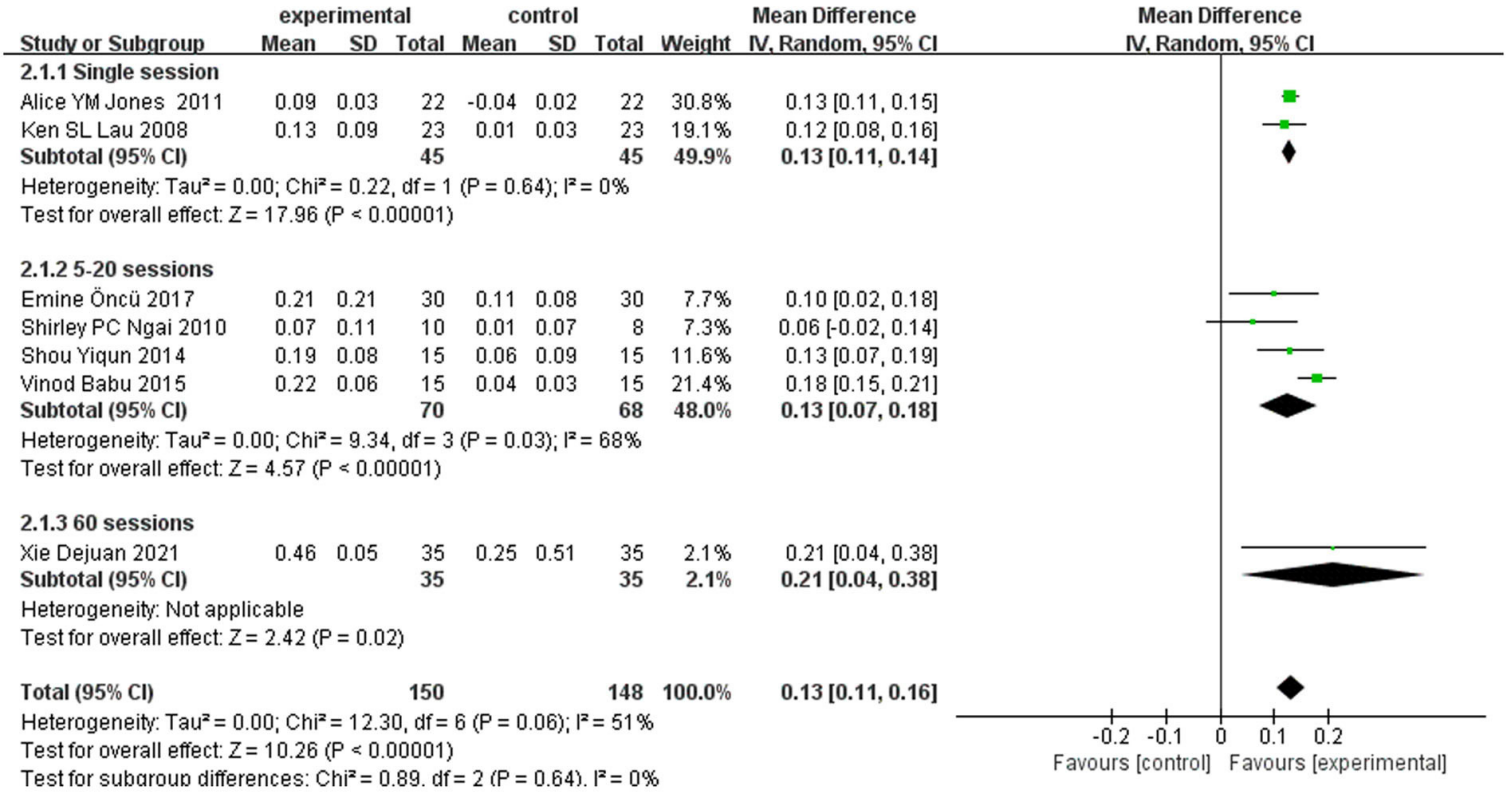
Subgroup analysis of FEV1 (course).

A total of seven (12, 15–18, 20, 21) studies reported FEV1 as an outcome. Due to the results of heterogeneity test (*I*^2^ = 51%, *P* = 0.06), a random-effect model was adopted. Meta-analysis showed that the experimental group can significantly increase FEV1 in patients with COPD [MD = 0.13 L, 95% CI (0.11–0.16), *P* < 0.00001]. As a rule of thumb, tests of funnel plot asymmetry should not be performed when fewer than 10 studies were included, because the power of tests was too low to distinguish chance from real asymmetry. To explore the possible sources of heterogeneity, subgroup analyses were conducted according to different intervention strategies (acu-TENS alone or combined with basic treatment, Figure 3) and the courses of treatment (single session, 5–20 sessions, or 60 sessions, Figure 4). Results showed that the therapeutic effect of acu-TENS on FEV1 may not be influenced by the intervention strategies and courses of intervention (test for subgroup differences: *P* all > 0.05).

However, subgroup analysis was not effective in reducing heterogeneity. Therefore, sensitivity analysis was performed by eliminating studies one by one to investigate the possible sources of heterogeneity. The sensitivity analysis of FEV1 showed that the heterogeneity was reduced (*I*^2^ = 0%, *P* = 0.54) after removing the article by Vinod et al. (15). A fixed-effect model was used to analyze the other six studies, and the result for FEV1 showed an improvement of 0.13 L in the experimental group compared with the control group [MD = 0.13 L, 95% CI (0.11–0.14), *P* < 0.00001].

#### FEV1% predicted

**Figure 5:**
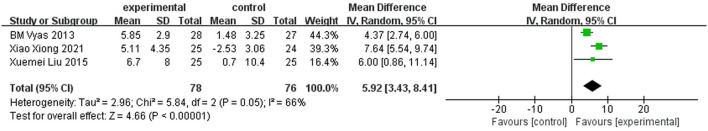
Forest plot of FEV1% predicted.

Among the included RCTs, three (13, 14, 19) reported FEV1% predicted as an outcome. Due to the obvious heterogeneous (*I*^2^ = 66%, *P* = 0.05), a random-effect model was used. Meta-analysis revealed that the treatment group can significantly increase FEV1% predicted in patients with COPD [MD = 5.92%, 95% CI (3.43–8.41), *P* < 0.00001] (Figure 5). The sensitivity analysis of FEV1% predicted showed that the heterogeneity was reduced (*I*^2^ = 0%, *P* = 0.56) after removing the article by Vyas et al. (14). A fixed-effect model was used to analyze the other two studies, and the result for FEV1% predicted showed an improvement of 7.41% in the experimental group compared with the control group [MD = 7.41, 95% CI (5.46–9.35), *P* < 0.00001].

#### 6-min walk distance

**Figure 6:**
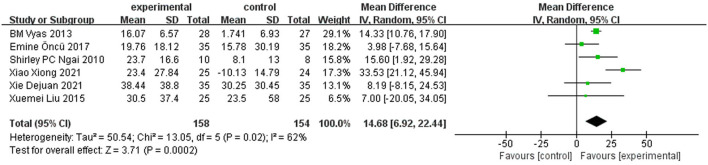
Forest plot of 6MWD.

A total of six trials (12–14, 17–19) reported 6-min walk distance (6MWD) as an outcome. The random-effect model was adopted according to the results of heterogeneity test (*I*^2^ = 62%, *P* = 0.02). Meta-analysis showed that the treatment group can significantly increase 6WMD in patients with COPD [MD = 14.68 m, 95% CI (6.92–22.44), *P* = 0.0002] (Figure 6). The sensitivity analysis of 6MWD showed that the heterogeneity was reduced (*I*^2^ = 0%, *P* = 0.48) after removing the article by Xiao et al. (13). A fixed-effect model was used to analyze the other five studies, and the result for 6MWD showed an improvement of 13.27 m in the experimental group compared with the control group [MD = 13.27 m, 95% CI (10.04–16.49), *P* < 0.00001].

#### Dyspnea visual analog scale

**Figure 7:**
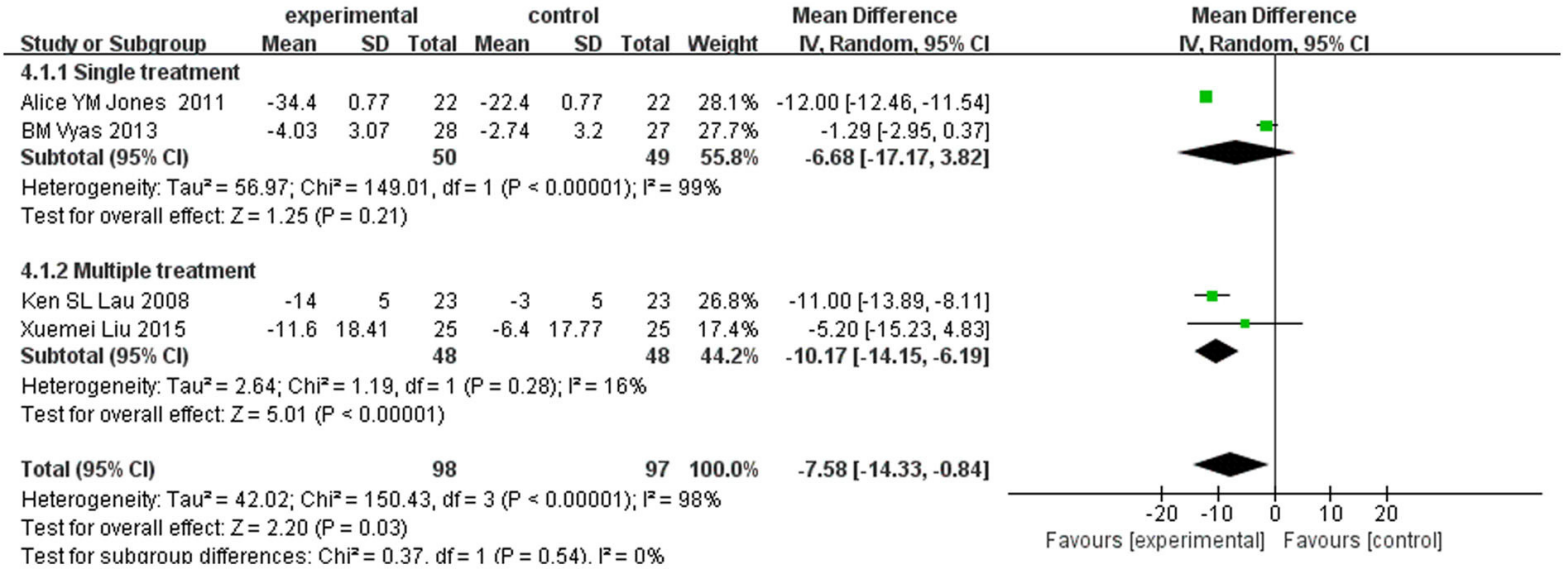
Subgroup analysis of DVAS.

Among the included studies, four (14, 19–21) reported dyspnea visual analog scale (DVAS) as an outcome. Because of the significant heterogeneity (*I*^2^ = 98%, *P* < 0.00001), a random-effect model was chosen. Meta-analysis showed that the treatment group can significantly decrease the scores of DVAS in patients with COPD [MD = −7.58, 95% CI (−14.33 to −0.84), *P* = 0.03] (Figure 7). Subgroup analysis of different courses suggested that there was no significant difference between subgroups (test for subgroup differences: *P* = 0.54).

However, subgroup analysis was not effective in reducing heterogeneity. Therefore, sensitivity analysis was performed, and the heterogeneity was reduced (*I*^2^ = 9%, *P* = 0.33) after removing the article by Vyas et al. (14). A fixed-effect model was used to analyze the other three studies, and the result for DVAS showed a statistical reduction of 11.96 points in the experimental group compared with the control group [MD = −11.96, 95% CI (−12.41 to −11.51), *P* < 0.00001].

#### Modified borg scale

**Figure 8:**
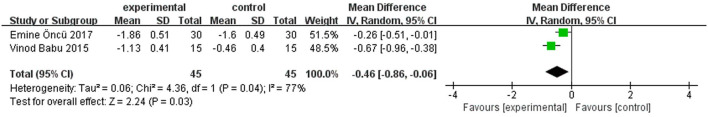
Forest plot of MBS.

Among the included studies, two (15, 17) reported the modified Borg scale (MBS) as an outcome. Due to the significant heterogeneity between studies (*I*^2^ = 77%, *P* = 0.04) and the inability to exclude individual studies for sensitivity analysis, a random-effect model was chosen. Meta-analysis showed that the experimental group can statistically decrease the scores of MBS in patients with COPD [MD = −0.46, 95% CI (−0.86 to −0.06), *P* = 0.03] (Figure 8).

#### COPD assessment test

**Figure 9:**
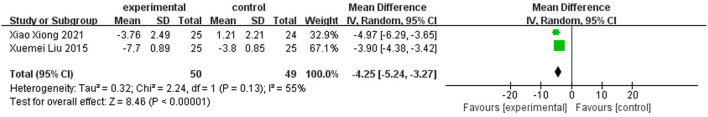
Forest plot of CAT.

The results of acu-TENS on COPD assessment test (CAT) were reported in two studies (13, 19). Due to the high heterogeneity between studies (*I*^2^ = 55%, *P* = 0.13) and the inability to exclude individual studies for sensitivity analysis, a random-effect model was adopted. Meta-analysis showed that the experimental group can significantly decrease the scores of CAT in patients with COPD [MD = −4.25, 95% CI (−5.24 to −3.27), *P* < 0.00001] (Figure 9).

#### Adverse effects

Among the included studies, five reported that no adverse events occurred (14, 18–21); one study reported that one patient had shoulder pain after acu-TENS (17); and the remaining studies did not mention adverse events (12, 13, 15, 16).

## Discussion

As we all know, systematic reviews and meta-analyses supply the highest levels of clinical evidence (22). In this review, the clinical evidence of acu-TENS in the treatment of COPD was systemically collected and evaluated for the first time to provide better guidance for clinical treatment. The main results indicated that acu-TENS was associated with a statistical improvement in pulmonary function, 6MWD, dyspnea, and the scores of the CAT in COPD patients. However, not all statistically significant differences are clinically relevant (23). The minimal clinically important difference (MCID) is the bridge between statistics and the clinical establishment and is the latest standard for determining the effectiveness of clinical trial interventions (24). Any amount of change that exceeds the MCID threshold is considered significant or important (25).

The pulmonary function test (PFT) is a common examination method in the respiratory department, which is crucial for diagnosis, evaluating therapeutic effects, and predicting perioperative risk and prognosis of COPD (26–28). One study reported that loss of lung function in older adults may lead to ventilation restriction during exercise, which reduces the accumulation of health benefits from physical activity (29). In this review, the level of changes in FEV1 exceeded the MCID of 100 ml (30), which suggested that these changes were clinically significant. Due to the lack of reported studies on the MCID value of FEV1% predicted, it was difficult to judge whether the statistical improvement of FEV1% predicted by acu-TENS is clinically significant. As a submaximal exercise test, the 6-min walk distance (6MWD) is mainly used to assess the physical performance of daily activities in patients with COPD (31). Studies have suggested that a 6MWD of less than 350 m or a decrease of 30 m or more within 1 year was significantly associated with higher mortality in patients with COPD (32, 33). Our results showed that the changes in 6MWD did not reach the MCID of 26 m (34), which indicated that these improvements in patients with COPD were insufficient evidence to support acu-TENS benefiting exercise capacity.

The COPD assessment test (CAT) is designed to evaluate the general health status of COPD patients and is widely used in clinical practice due to its excellent measurement properties (35). The meta-analysis showed that acu-TENS was associated with a statistically significant reduction in the score of CAT. Furthermore, the overall effect size of CAT exceeded the MCID by 2 points (36), which suggested that acu-TENS therapy has clinical benefits on the health status of patients with COPD. Dyspnea is one of the most common and distressing symptoms of various pulmonary diseases. Research has found that the severity of dyspnea is significantly related to decreased quality of life and survival rate (37). In this review, acu-TENS showed statistically significant improvement in dyspnea assessed by the MBS and DVAS. However, it is important to note that Ekstrom et al. (38) pointed out that an improvement of about 10 mm in a 100 mm VAS was clinically significant for chronic dyspnea, and the overall effect size of DVAS in this analysis was lower than the MCID of 10 mm. As for the MCID of MBS, there is still a lack of relevant research reports. In this context, we concluded that acu-TENS has little effect on the improvement of dyspnea in patients with COPD.

The TENS device is a hand-held, battery-powered machine that delivers pulsed currents by using electrode pads (39). Based on the acupuncture-like efficacy of TENS (40), researchers placed TENS electrodes on acupoints, known as acu-TENS. As a noninvasive and cost-efficient option, acu-TENS has become a common alternative therapy to acupuncture (41). Our meta-analysis showed that acu-TENS treatment can improve the pulmonary function indicators and health status in COPD patients with minimal benefit on exercise capacity and dyspnea. Acupuncture and acu-TENS are two different forms of acupoint stimulation therapy, but the results of studies on their efficacy in patients with COPD are not completely consistent. According to the review by Wang et al. (42), acupuncture can not only improve the pulmonary function index of patients with COPD but also improve their exercise ability and alleviate breathlessness. However, the degree of improvement in dyspnea and exercise capacity in our meta-analysis was not clinically significant, thus further confirmation is still needed. Ngai et al. (18) found that β-endorphin levels increased significantly in the acu-TENS group after treatment but not in the placebo-TENS and sham-TENS groups, suggesting that the release of opioids induced by TENS may depend on acupoint stimulation. Furthermore, research has found that stimulation of opioid receptors could enhance the relaxation of canine airway smooth muscle mediated by beta-adrenal receptors (43). This may be one of the mechanisms of acu-TENS in the treatment of COPD. However, the mechanisms of acu-TENS still need further study.

## Limitations and implications

In this review, some deficiencies should not be ignored. First, only a small number of RCTs were included, and they were all single-center and small-sample studies. Second, due to the incomplete information reported by studies and the deficiencies in study design, the methodological quality of included studies was generally low. Third, since few studies were included, no publication bias was tested. However, the possibility of publication bias cannot be excluded since none of the trials were publicly registered. Fourth, it is important to ensure that the acu-TENS is safe. However, about half of the studies did not report adverse reactions, which affected the judgment of safety. Fifth, since no studies have reported follow-up, the long-term efficacy of acu-TENS is unclear.

This meta-analysis found several typical problems in this study, and addressing these problems will be of great help to enhance the quality of TCM research. First, although RCTs are the best method to evaluate the efficacy of interventions, low-quality RCTs can lead to incorrect assessments. Thus, future research on acu-TENS should focus on the registration of clinical trials, which can make research information more transparent, reduce publication bias, and improve the credibility of TCM research. Second, as the incomplete research report is one of the reasons for the waste of TCM research, we suggest that future research on acu-TENS should be reported in accordance with the statement of CONSORT (44). Finally, the occurrence of adverse reactions is related to the lack of enough attention to safety. Therefore, more attention should be paid to the safety of acu-TENS in future studies.

## Conclusion

Considering the available data, this meta-analysis concluded that acu-TENS seems to be effective in improving pulmonary function and health status in patients with COPD, with little effect on exercise capacity and dyspnea. However, the results should be interpreted with caution, limited by the number and methodological quality of the included studies, and high-quality RCTs are needed in the future to investigate the efficacy and safety of acu-TENS.

## Data availability statement

The original contributions presented in the study are included in the article/supplementary material, further inquiries can be directed to the corresponding author.

## Author contributions

YW and YD: design, investigation, manuscript writing, and supervision. LW and JD: methodology, verification, and analysis. NY: verification, writing, and editing. All authors contributed to the article and approved the submitted version.

## Conflict of interest

The authors declare that the research was conducted in the absence of any commercial or financial relationships that could be construed as a potential conflict of interest.

## Publisher's note

All claims expressed in this article are solely those of the authors and do not necessarily represent those of their affiliated organizations, or those of the publisher, the editors and the reviewers. Any product that may be evaluated in this article, or claim that may be made by its manufacturer, is not guaranteed or endorsed by the publisher.
